# Genome-Wide Identification, Characterization, and Expression Analysis of *BBX* Genes During Anthocyanin Biosynthesis in Mango (*Mangifera indica* L.)

**DOI:** 10.3390/biology14080919

**Published:** 2025-07-23

**Authors:** Chengkun Yang, Muhammad Mobeen Tahir, Yawen Zhang, Xiaowen Wang, Wencan Zhu, Feili Li, Kaibing Zhou, Qin Deng, Minjie Qian

**Affiliations:** 1 Key Laboratory of Quality Regulation of Tropical Horticultural Crop in Hainan Province, School of Tropical Agriculture and Forestry, Hainan University, Haikou 570228, China; hndxyck@hainanu.edu.cn (C.Y.); mubeentahir924@gmail.com (M.M.T.); 24110901000096@hainanu.edu.cn (Y.Z.); xiaowenwang@hainanu.edu.cn (X.W.); wencanzhu@hainanu.edu.cn (W.Z.); feili.li@hainanu.edu.cn (F.L.); zkb@hainanu.edu.cn (K.Z.); 2 Sanya Institute of Breeding and Multiplication, Hainan University, Haikou 570228, China

**Keywords:** B-box protein, gene expression, light-responsive

## Abstract

B-box (BBX) transcription factors are critical regulators of light-mediated anthocyanin biosynthesis, influencing peel coloration in plants. To explore their role in red mango cultivars, we identified 32 *BBX* genes (*MiBBX1*–*MiBBX32*) in the mango (*Mangifera indica* L.) genome using a genome-wide analysis. Phylogenetic and structural analyses classified these genes into five subfamilies based on conserved domains. A collinearity analysis revealed segmental duplication as the primary mechanism of *MiBBX* gene family expansion, with purifying selection shaping their evolution. A promoter analysis identified numerous light- and hormone-responsive *cis*-elements, indicating regulatory roles in the light and hormonal signaling pathways. Expression profiling in the ‘Sensation’ cultivar revealed organ-specific patterns, with several *MiBBX* genes showing higher expression in the peel than in the flesh. Many of these genes also consistently exhibited elevated expression in the peel of red-skinned cultivars (‘Sensation’ and ‘Guifei’) compared to yellow and green cultivars, suggesting their role in red peel pigmentation. Furthermore, postharvest light treatment of ‘Hongmang No. 6’ fruit significantly upregulated multiple *MiBBX* genes, suggesting their involvement in light-induced anthocyanin accumulation in red mango peel. These findings provide valuable insights into the molecular mechanisms governing light-regulated peel coloration in mango and establish a foundation for functional studies of *MiBBX* genes in fruit pigmentation.

## Simple Summary

Mango is a popular tropical fruit appreciated for its rich flavor and vibrant appearance. Mango peel exhibits diverse colors, including red, green, and yellow. The formation of red peel primarily depends on anthocyanins. Anthocyanin biosynthesis is influenced by light, a process regulated by B-box (BBX) proteins. This study identified 32 *MiBBX* genes from the mango genome and conducted a comprehensive analysis. An analysis of the mango *BBX* gene promoter regions indicated their potential roles in light and hormonal signaling pathways. An organ-specific expression analysis revealed that several *MiBBX* genes showed higher expression in the peel of the red-skinned mango ‘Sensation’. Multiple genes exhibited higher expression in the peel of red varieties compared to the yellow or green varieties. Furthermore, several *MiBBX* genes were upregulated in the peel under postharvest light exposure. These findings indicate that specific *MiBBX* genes are likely involved in regulating anthocyanin biosynthesis and contribute to red peel coloration in mango. This study establishes a foundation for the future functional characterization of *MiBBX* genes in fruit pigmentation.

## 1. Introduction

Zinc-finger proteins represent one of the largest classes of transcription factors in plants, characterized by their conserved zinc-binding motifs that stabilize the tertiary protein structure [1]. Among them, B-box (BBX) proteins constitute a distinct subfamily that play critical regulatory roles in numerous biological processes, including photomorphogenesis, flowering time control, hormonal signaling, and responses to abiotic and biotic stresses [2,3].

Structurally, plant BBX proteins typically contain one or two B-box domains at the N-terminus, which are involved in protein–protein interactions. Furthermore, some members additionally harbor a CCT (CONSTANS, CO-like, and TOC1) domain at the C-terminus, contributing to nuclear localization and transcriptional regulation. Based on the presence and combination of B-box and CCT domains, BBX proteins are generally classified into five distinct subfamilies [4,5].

The first identified BBX gene, CONSTANS (*AtBBX1*), from *Arabidopsis thaliana*, was shown to regulate photoperiodic flowering [6]. With the release of numerous plant genome sequences, BBX gene families have been identified in various species through genome-wide analyses, including *Arabidopsis* [4], maize (*Zea mays*) [7], tomato (*Solanum lycopersicum*) [8], apple (*Malus domestica*) [9], pear (*Pyrus bretschneideri*) [10], rice (*Oryza sativa*) [5], and *Dendrobium officinale* [11]. These studies have primarily focused on the gene structure, classification, and expression patterns, revealing both conserved and species-specific features of BBX genes. However, functional characterization through genetic manipulation has so far been reported mostly in *Arabidopsis* [12]. In addition to their classical role in flowering regulation [6], *BBX* genes have been associated with seedling development [13], shade avoidance [14], pollen growth [10], heat stress response [15], and brassinosteroid and light signaling transduction [16].

Anthocyanins are important pigments, contributing to fruit coloration, antioxidant activity, and commercial value in many fruit crops [17,18]. They are synthesized via the flavonoid biosynthetic pathway, which includes enzymes such as chalcone synthase (CHS), flavanone 3-hydroxylase (F3H), dihydroflavonol 4-reductase (DFR), and UDP-glucose:flavonoid 3-O-glucosyltransferase (UFGT), and they are regulated by MYB-bHLH-WD40 transcription factor complexes [18]. The biosynthesis and accumulation of anthocyanins are tightly regulated by environmental cues, including light and temperature [18,19]. In addition to their role in pigmentation, anthocyanins serve as antioxidants and play protective roles against various abiotic and biotic stresses [17,18,19]. Notably, anthocyanin biosynthesis is a well-characterized model system for studying transcriptional regulation in plants, featuring complex networks of activators and repressors that respond dynamically to environmental and developmental cues [20]. This regulatory framework provides key insights into gene regulation mechanisms and transcription factor interactions governing pigment production.

Recent studies highlight that BBX proteins can act as key regulators of light-mediated anthocyanin biosynthesis by interacting with transcription factors such as ELONGATED HYPOCOTYL 5 (HY5) and v-Myb Avian Myeloblastosis Viral Oncogene Homolog (MYB), thereby modulating anthocyanin biosynthetic genes [21,22,23]. In apple, functional evidence supports the role of specific BBX members in light-regulated pigmentation. For example, MdBBX21 positively regulates light-induced anthocyanin accumulation in apple peel by activating anthocyanin biosynthetic genes [24]. Similarly, MdBBX20 integrates ultraviolet radiation and low-temperature signals to promote anthocyanin biosynthesis [25]. Additionally, studies such as “Interaction between UV-B and plant anthocyanins” provide insight into the environmental responsiveness of this regulation. While BBX involvement has also been suggested in other fruits, such as pear [26,27,28], its comprehensive functional validation is still limited.

Mango (*Mangifera indica* L.), often referred to as “the king of fruits,” is a commercially important tropical fruit widely cultivated in India, China, Thailand, and other regions. Its attractive appearance, rich flavor, and nutritional value make the fruit quality a major focus of breeding and postharvest research [29,30]. Peel coloration, largely determined by anthocyanin accumulation, is one of the most visually important fruit quality traits influencing consumer preference. Although anthocyanin biosynthesis in mango has been previously reported [31,32,33], the regulatory roles of BBX transcription factors in light-mediated peel coloration remain largely unexplored.

With the availability of the mango reference genome [34,35], it is now feasible to conduct a comprehensive genome-wide analysis of the mango *BBX* (*MiBBX*) gene family. In this study, we systematically identified *BBX* genes in the mango genome and analyzed their phylogenetic relationships, gene structures, conserved domains, promoter *cis*-elements, and expression profiles across various mango organs. We further analyzed the organ-specific expression patterns of *MiBBX* genes, with a particular focus on their response to light treatment during postharvest fruit storage. Our results provide novel insights into the potential regulatory functions of BBX transcription factors in mango peel coloration and lay a foundation for future functional studies aimed at improving mango fruit quality through molecular breeding.

## 2. Materials and Methods

### 2.1. Identification of the MiBBX Gene Family

The sequence of the BBX protein of *Arabidopsis thaliana* was downloaded from the Tair database (https://www.arabidopsis.org/, accessed on 4 March 2021), and the mango genome was obtained from the BIG Genome Warehouse (https://ngdc.cncb.ac.cn/gwh/, accessed on 4 March 2021) under accession number PRJCA002248 [4,35]. First, all CDS sequences from the mango genome were extracted using TBtools software v1.09868 and translated into protein sequences [36]. Using AtBBX proteins as query sequences, TBtools was then employed to search for putative MiBBX proteins [36]. The initially screened protein sequences were subsequently aligned against the SwissProt database using the NCBI-BLASTP tool to remove redundant entries (https://blast.ncbi.nlm.nih.gov/, 8 March 2021), and the parameters were set to their default values [37]. Next, the candidate protein sequences were further verified for conserved domains using NCBI Batch CD-Search (the parameters were set to their default values) [38]. The protein length, isoelectric point (PI), molecular weight (MW), grand average of hydropathicity, instability index, and aliphatic index values of the predicted MiBBX proteins were determined using ExPASy (https://www.expasy.org/, 8 March 2021) [39]. Subcellular localization was predicted using Plant-mPLoc (http://www.csbio.sjtu.edu.cn/bioinf/plant-multi/, 8 March 2021) [40].

### 2.2. Phylogenetic, Gene Structure, and Conservation Analysis of MiBBXs

Full-length BBX protein sequences from mango and *Arabidopsis* were aligned using Clustal X [41]. The phylogenetic tree of BBX proteins was constructed using the maximum likelihood (ML) method in IQ-TREE2, with automatic model selection, 1000 bootstrap replicates, and default settings for all other parameters [42]. Conserved domains were predicted using NCBI CD-Search [38] and Pfam (the parameters were set to their default values) [43], while conserved motifs of mango were identified using the MEME-suite (http://meme-suite.org/tools/meme, 8 March 2021) (the parameters were set to their default values) [44]. The gene structures, conserved motifs, domains, and sequence logos were visualized using TBtools [36].

### 2.3. Chromosomal Distribution, Collinearity, and Selective Pressure Analysis

Chromosomal locations of *MiBBX* genes were determined based on the mango genome annotation. A collinearity analysis was performed using TBtools and MCscanX 1.0 [36]. The gene density maps and chromosomal locations of *MiBBX* genes were visualized using TBtools [36]. The nonsynonymous (*Ka*) and synonymous (*Ks*) substitution rates for homologous gene pairs (both between species and within species) were calculated using the NG (Nei–Gojobori) algorithm within TBtools to assess the selective pressure (*Ka*/*Ks*) [36].

### 2.4. Promoter Cis-Acting Element Analysis

The 2000 bp genomic sequences upstream of the translation start site (ATG) for each *MiBBX* gene were extracted as putative promoter regions. Cis-acting regulatory elements were predicted using the PlantCARE database (http://bioinformatics.psb.ugent.be/webtools/plantcare/html/, 8 March 2021) [45].

### 2.5. Expression Analysis of MiBBXs Using RNA-Seq Data

Publicly available mango RNA-seq data (NCBI BioProject: PRJNA487151) were downloaded [34]. The transcript abundance was quantified as Transcripts Per Kilobase Million (TPM) using Kallisto [46]. The analyzed samples included ‘Alphonso’ mango (root, bark, mature leaf, flower, peel, flesh, and seed); peel and flesh tissues of the red cultivar ‘Sensation’ at 94, 100, 106, and 112 days after full bloom (DAFB); and peel tissues of ‘Sensation’ (red), ‘Hongyu’ (yellow), and ‘Guire-82’ (green) cultivars at immature (green mature, ~100 DAFB) and mature (full-ripe, ~120 DAFB) developmental stages.

### 2.6. Fruit Sampling and RT-qPCR Analysis

Fruits of the red cultivar ‘Guifei’ were collected at green mature, mid-ripe, and full-ripe stages from the Hainan Fruit Island Agricultural Development Co., Ltd. (Sanya, China). After harvesting, the fruits were washed; then, the peel and flesh tissues were separated using a sharp knife.

Bagged physiologically mature fruits of ‘Hongmang No. 6’ were collected from the South Subtropical Crops Research Institute, Chinese Academy of Tropical Agricultural Sciences. The control fruits were stored in darkness, while the treated fruits were exposed to simulated sunlight conditions (4.5 μW·cm^−2^ UV-B + 16 W·m^−2^ white light) at 17 °C and 80% relative humidity. Peel samples from the treated fruits were collected at 0, 6, 24, 72, 144, and 240 h post-treatment for RT-qPCR analysis, following protocols from our previous study [47].

Total RNA was extracted using the DP441 kit (TIANGEN, Beijing, China). First-strand cDNA synthesis was performed using the HiScript III 1st Strand cDNA Synthesis Kit (Vazyme, R312, Nanjing, China). Reverse transcription quantitative PCR (RT-qPCR) was conducted using ChamQ Universal SYBR RT-qPCR Master Mix (Vazyme, Q711), following the manufacturers’ protocols. We used Primer3 to design *MiBBX* gene primers, using the CDS of the aforementioned mango samples as the template (https://primer3.ut.ee/ 28 March 2021). The mango *Actin* gene was used as the internal reference [48]. The primer sequences are listed in Supplementary Table S1. The relative gene expression levels were calculated using the 2^−ΔΔCt^ method [49]. Data visualization was performed using GraphPad Prism 9.5.

### 2.7. Statistical Analysis

A one-way ANOVA followed by Tukey’s multiple range test was conducted using SPSS 26.0 (SPSS Inc., Chicago, IL, USA) to analyze the RT-qPCR data from the ‘Guifei’ peel and flesh tissues separately. Differences were considered statistically significant at *p* < 0.05 and are indicated by different lowercase letters. Independent-sample *t*-tests were applied to compare the gene expression between the peel and flesh tissues of ‘Guifei’ at the same developmental stage, as well as between the treated and control samples of ‘Hongmang No. 6’ at each time point. The significance levels are indicated as * (*p* < 0.05), ** (*p* < 0.01), and *** (*p* < 0.001).

## 3. Results

### 3.1. Identification of BBX Genes in Mango

A total of 32 *BBX* genes were identified from the mango genome and designated as *MiBBX1* to *MiBBX32*. The comprehensive information for each gene, including the gene ID, chromosome location, coding sequence (CDS) length, protein length, isoelectric point (pI), MW, instability index, aliphatic index, grand average of hydropathicity, and predicted subcellular localization, is provided in Supplementary Table S2. The CDS length of *MiBBX* genes ranged from 492 bp (*MiBBX7*) to 1536 bp (*MiBBX11*), corresponding to predicted protein lengths between 163 and 511 (Supplementary Table S2). The MWs of these proteins varied from 18.04 to 56.73 kDa, while the predicted pI values ranged from 4.79 (MiBBX7) to 9.17 (MiBBX20) (Supplementary Table S2). The aliphatic index values ranged from 56.43 (MiBBX13) to 77.47 (MiBBX28). Among all identified MiBBX proteins, MiBBX22, MiBBX23, and MiBBX26 were predicted to be the most stable, with an instability index lower than 40, whereas the remaining proteins were predicted as unstable. All MiBBX proteins were predicted to be hydrophilic in nature and localized to the nucleus, suggesting their possible role in transcriptional regulation.

### 3.2. Gene Structure, Conserved Domain, and Phylogenetic Analysis of MiBBXs

**Figure 1:**
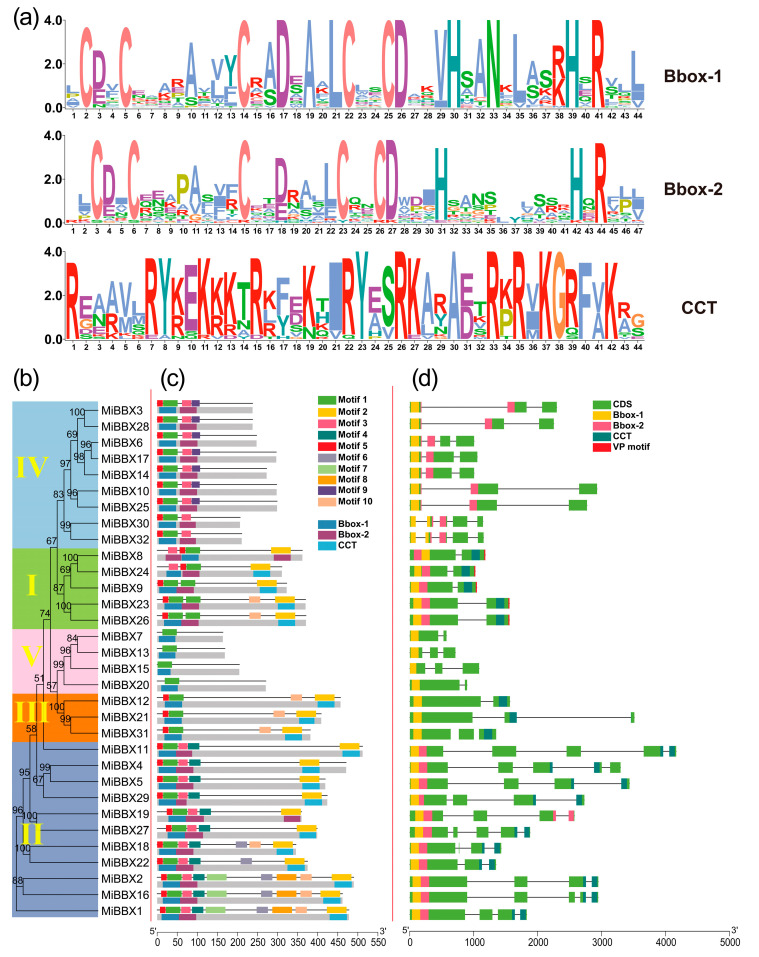
SeqLogos, phylogenetic relationships, conserved domains, motifs, and genetic structure analysis of MiBBXs. (**a**) SeqLogo representations were generated based on the conserved domain amino acid sequences of MiBBXs using Clustal X alignment. (**b**) Phylogenetic relationships of MiBBXs. Members of different subgroups are color-coded. (**c**) Conserved domains and motif compositions of MiBBXs. The upper section for each member displays conserved domain information, while the lower section shows motif patterns. Motifs 1–10 are presented in distinctively colored boxes. (**d**) Exon–intron structures of *MiBBX* genes. Green boxes represent exons; black lines denote introns. Regions encoding conserved domains are annotated on the gene structures in yellow (B-box1), pink (B-box2), blue (CCT domain), and crimson (VP motif). Protein and nucleotide sequence lengths can be estimated using the scale bar at the bottom.

The conserved motifs of the two B-box domains (B-box 1 and B-box 2) and the CCT domain were analyzed, and their motif logos are presented in Figure 1a. Although the amino acid distributions in B-box 1 and B-box 2 were largely similar, some differences were observed. Generally, both domains contained five cysteine (Cys) residues, with four of them arranged in a characteristic Cys-X-X-Cys motif. In most cases, the second Cys-X-X-Cys motif was flanked by leucine (L) and aspartic acid (D) residues. Additionally, several other conserved residues, such as histidine (H), alanine (A), and asparagine (N), were identified in both B-box domains. The CCT domain displayed a high abundance of arginine (R) and lysine (K), followed by tyrosine (Y) and alanine (A), indicating its highly conserved nature across the gene family. (Figure 1a).

To further investigate the evolutionary relationships among MiBBX proteins, a phylogenetic tree was constructed using full-length BBX protein sequences from mango and *Arabidopsis* (Supplementary Figure S1, Figure 1b). Based on the phylogenetic analysis, the MiBBX proteins were clustered into five distinct clades, namely, Clades I to V. Clade I comprised five MiBBX members that contained the Bbox-1, Bbox-2, and CCT domains and a VP motif with the conserved core sequence G-I/V-V-P-S/T-F. Clade II consisted of eleven MiBBX genes, which also harbored Bbox-1, Bbox-2, and CCT domains; however, the Bbox-2 domain in this clade exhibited considerable structural divergence compared to Clade I, characterized by the presence of motifs 3 and 4 instead of motif 1. Clade III included members that lacked one of the Bbox domains, whereas Clade IV contained proteins missing the CCT domain and possessing a distinct Bbox-2 domain composed of motifs 3 and 9. Clade V consisted of proteins that contained only a single Bbox domain (Figure 1b–d). The gene structure analysis further revealed that most *MiBBX* genes consisted of two to five exons and one to four introns, with genes belonging to the same clade generally exhibiting similar exon–intron organizations, indicating their close evolutionary relationships (Figure 1d).

### 3.3. Chromosomal Distribution, Collinearity, and Selective Pressure

**Figure 2:**
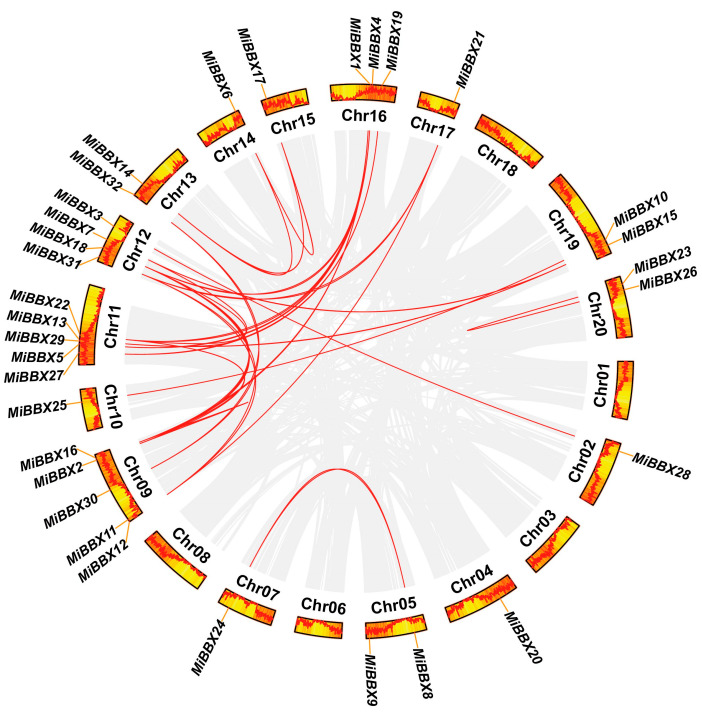
Schematic representation of *MiBBX* chromosomal distribution and interchromosomal relationships. Gray lines depict all syntenic blocks across the mango genome, while red lines highlight collinear *BBX* gene pairs. Chromosome numbers are indicated at the base of each chromosome. The colors within the ring represent the varying levels of gene density, ranging from red to yellow. The farther the red line is from the center of the circle, the higher the gene density is.

The 32 *MiBBX* genes were unevenly distributed across 15 chromosomes (Chr), with gene numbers per chromosome ranging from one to five. No *MiBBX* genes were detected on Chr01, Chr03, Chr04, Chr06, Chr08, or Chr18. Most *MiBBX* genes were in regions with a high gene density (Figure 2). A total of 26 segmental duplication events were identified; however, no tandem duplications involving *MiBBX* genes were observed (Figure 2, Supplementary Table S3). Collinearity analysis with other plant species revealed no syntenic *MiBBX* gene pairs with the monocots maize or rice, while 15, 27, 26, and 49 syntenic gene pairs were identified between mango and the eudicots *Arabidopsis*, citrus, grape, and apple, respectively (Supplementary Figure S2, Supplementary Table S3), reflecting closer phylogenetic relationships. The majority of these collinear gene pairs exhibited Ka/Ks ratios significantly less than 1 (Supplementary Table S3), suggesting that they have predominantly undergone purifying selection during evolution.

### 3.4. Analysis of Cis-Regulatory Elements in the Promoter Region of MiBBX Genes

**Figure 3:**
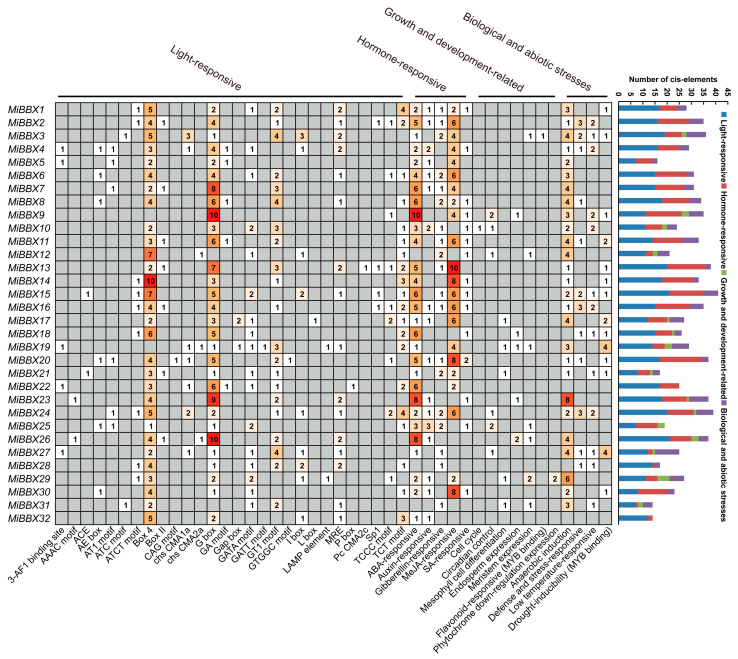
Analysis of *cis-*acting elements in the promoter regions of the 32 *MiBBX* genes. The heatmap region depicts the abundance of *cis-*regulatory elements, where the shade of red and black numbers indicates the number of elements, while gray denotes the absence of the element type in question. The bar chart illustrates the statistical distribution of different element types per promoter.

A total of 913 putative cis-acting elements were identified in the promoter regions of the *MiBBX* genes. Light-responsive elements (457, 50.05%) and hormone-responsive elements (285, 31.22%) were the most abundant categories. Notably, G-box elements, which serve as binding sites for the photomorphogenesis transcription factor HY5, were present in the promoters of most *MiBBX* genes. Several promoters also contained MYB recognition element (MRE) elements, potential binding sites for MYB transcription factors involved in the regulation of anthocyanin biosynthesis, suggesting potential roles for *MiBBXs* in light response and anthocyanin regulation in mango. Additionally, many promoters harbored abscisic acid (ABA)-responsive elements (ABRE) and methyl jasmonic acid (MeJA)-responsive elements (CGTCA motifs and TGACG motifs), indicating potential involvement in hormone signaling pathways. However, no clear correlation was observed between the type or number of *cis*-elements and the gene subfamily classification of *MiBBX* genes (Figure 3).

### 3.5. Organ-Specific Expression Profiles of MiBBXs in ‘Sensation’ Mango

**Figure 4:**
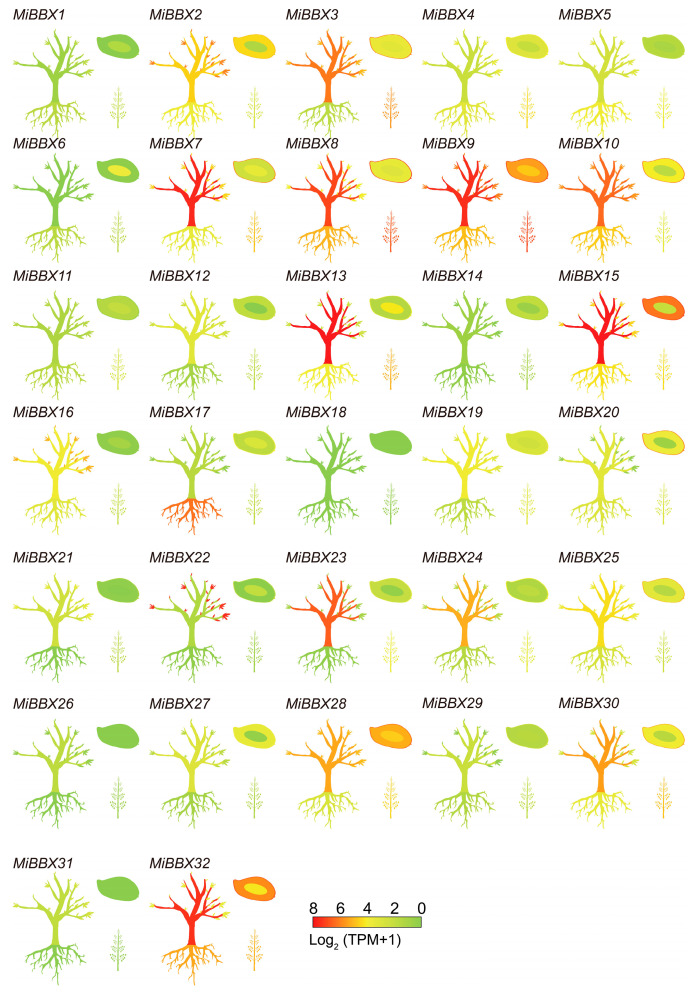
The expression profiles of *MiBBXs* in the roots, mature bark, mature leaves, peels, pulp, flowers, and seeds of ‘Sensation’, ranging from green (low) to red (high).

The *MiBBX* genes exhibited distinct organ-specific expression patterns in ‘Sensation’ mango (Figure 4). Notably, the bark showed high expression for several genes, including *MiBBX3* (TPM = 69.64), *MiBBX7* (TPM = 180.16), *MiBBX8* (TPM = 128.15), and *MiBBX9* (TPM = 165.60), among others. Mature leaves displayed elevated expressions of *MiBBX2*, *MiBBX3*, *MiBBX9*, and *MiBBX22*, while MiBBX17 was uniquely and strongly expressed in the roots (TPM = 75.69). In addition, MiBBX3, MiBBX8, and *MiBBX9* were prominent in the flowers, and a subset of genes, including *MiBBX3*, *MiBBX7*, *MiBBX8*, *MiBBX9*, *MiBBX15*, *MiBBX28*, and *MiBBX32*, showed high expression in the fruit peel and/or flesh, with *MiBBX9* and *MiBBX28* also elevated in seeds. Conversely, *MiBBX1* and *MiBBX14* exhibited low or negligible expression across most organs.

### 3.6. Expression of MiBBXs in the Peel vs. Flesh of Red Cultivars

**Figure 5:**
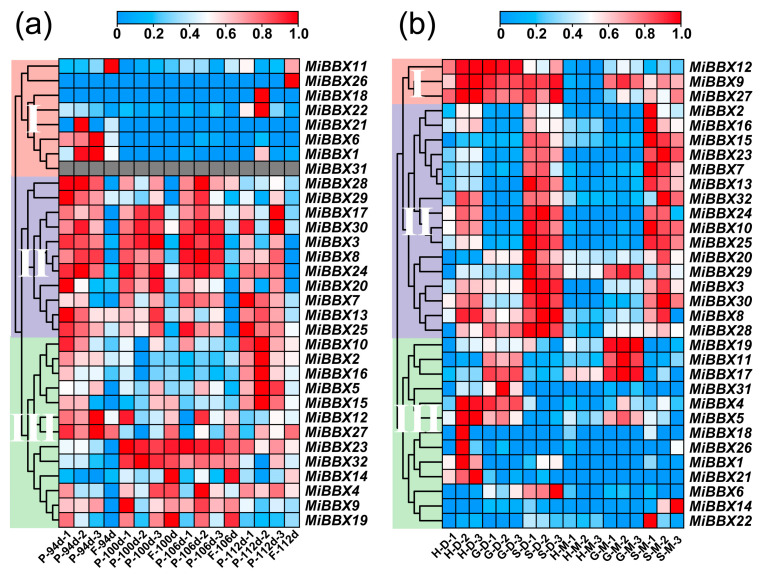
(**a**) The expression profiles of *MiBBXs* in the peel and pulp tissues of ‘Sensation’ at 94, 100, 106, and 112 DAFB. P: peel; F: flesh. (**b**) The expression profiles of *MiBBXs* in the peel tissues of ‘Sensation’ (red), ‘Hongyu’ (yellow), and ‘Gure-82’ (green) in the dark-green ripe, moderately ripe, and fully ripe fruit, ranging from blue (low) to red (high). H: ‘Hongyu’ mango; G: ‘Guire-82’ mango; S: ‘Sensation’ mango; D: development stage; M: mature stage. The numbers represent biological replicates.

For instance, *MiBBX8* TPM values in the peel were 6.94-, 3.54-, 4.92-, and 5.42-fold higher than in the flesh at 94, 100, 106, and 112 DAFB, respectively. In red mango cultivars, anthocyanins primarily accumulate in the peel due to direct light exposure, while little accumulation occurs in the flesh [34]. An analysis of *MiBBX* gene expression in the peel and flesh of ‘Sensation’ revealed three expression patterns: Type I genes (eight members) exhibited negligible or low expression across all developmental stages. Type II genes (eleven members) exhibited relatively high expression, consistently higher in the peel than the flesh at each stage. For example, the expression of *MiBBX8* was 6.94-, 3.54-, 4.92-, and 5.42-fold higher in peel compared to flesh at 94, 100, 106, and 112 DAFB, respectively, while *MiBBX24* showed corresponding fold changes of 5.64, 2.79, 5.37, and 9.39. Type III genes also displayed relatively high expression, generally higher in the peel but with smaller and stage-dependent fold differences; for instance, *MiBBX10* expression in the peel was 1.90-, 1.14-, 1.46-, and 1.56-fold higher than in the flesh at the respective stages (Figure 5a, Supplementary Table S4). All fold changes were calculated based on normalized TPM values (Figure 5a, Supplementary Table S4).

Genes involved in anthocyanin biosynthesis typically exhibit higher expression in red cultivars (with red peel at immature/mature stages) compared to yellow (green immature, yellow mature) or green (green at both stages) cultivars [34]. Consistently, *MiBBX* expression patterns in the peel of ‘Sensation’ (red), ‘Hongyu’ (yellow), and ‘Guire-82’ (green) grouped into three categories: Type I genes (three members: *MiBBX9*, *MiBBX12*, and *MiBBX27*) showed relatively high expression across cultivars and stages, with the highest expression in ‘Guire-82’ and ‘Sensation’ at maturity. Type II genes (sixteen members) exhibited high expression, consistently highest in the red cultivar ‘Sensation’. For example, *MiBBX25* expression in immature ‘Sensation’ peel was 5.14- and 43.13-fold higher than in ‘Hongyu’ and ‘Guire-82’, respectively; these differences further increased to 141.44- and 75.25-fold in the mature stage. Type III (thirteen members) showed low overall expression without distinct cultivar-dependent patterns (Figure 5b, Supplementary Table S5).

### 3.7. Expression of MiBBXs in the Peel of Different Colored Cultivars

**Figure 6:**
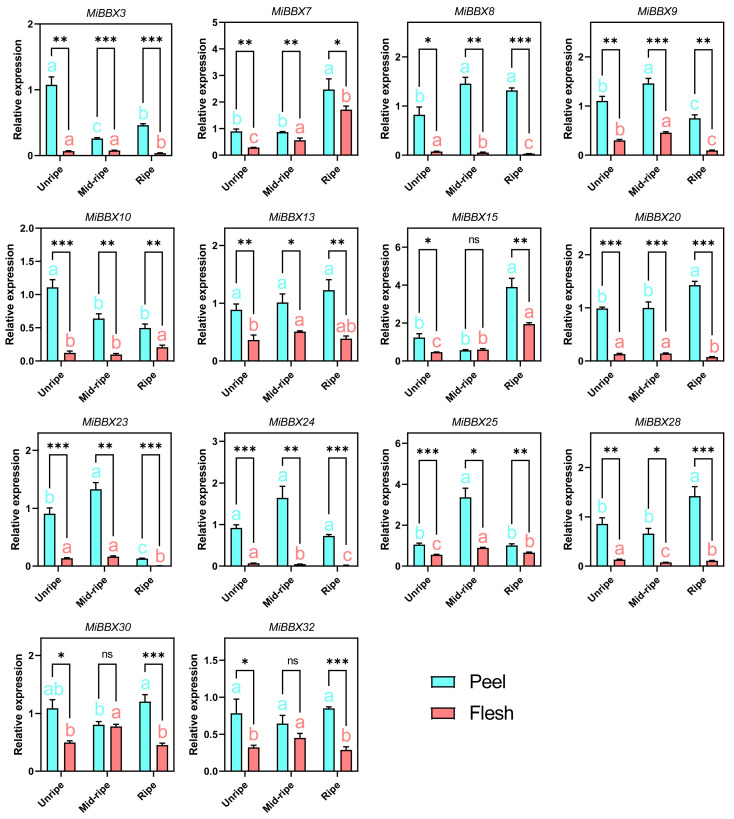
RT-qPCR analysis of *MiBBXs* in the peels and pulp of ‘Guifei’ at the unripe, mid-ripe, and ripe stages. Statistical significance (determined by Student’s *t*-test) is indicated as follows: * *p* < 0.05, ** *p* < 0.01, *** *p* < 0.001, ns *p* > 0.05. Unripe: the green mature stage of the fruit; Mid-ripe: the mid-ripe stage of the fruit; Ripe: the full-ripe stage of the fruit. Columns labeled with different letters represent samples with significantly different values, *p* < 0.05, determined by one-way ANOVA, followed by Tukey’s tests. Data at three development stage of peel (marked with sky blue letters) or flesh (marked with red letters) samples were tested separately.

To assess the expression consistency across red cultivars, RT-qPCR was performed on 14 Type II/III genes (*MiBBX3*, *MiBBX7*, *MiBBX8*, *MiBBX9*, *MiBBX10*, *MiBBX13*, *MiBBX15*, *MiBBX20*, *MiBBX23*, *MiBBX24*, *MiBBX25*, *MiBBX28*, *MiBBX30*, and *MiBBX32*) in ‘Guifei’ peel and flesh at green mature (~‘Sensation’ 94 DAFB), mid-ripe, and full-ripe (~‘Sensation’ 112 DAFB) stages. The expression was significantly higher in the peel than in the flesh for nearly all genes at each stage, except *MiBBX5*, *MiBBX30*, and *MiBBX32* at the mid-ripe stage. For example, *MiBBX3* peel expression was 16.03, 3.34, and 12.23 times higher than in the flesh at the green mature, mid-ripe, and full-ripe stages, respectively, while the expression levels of *MiBBX24* were 13.42-, 35.90-, and 40.96-fold higher. This pattern mirrored that of the ‘Sensation’ expression. Some genes also showed ripening-related trends: *MiBBX7* increased in both tissues; *MiBBX10* decreased in the peel but increased in the flesh; *MiBBX20* showed the opposite trend to *MiBBX10* (Figure 6). Given that anthocyanin accumulation occurs in the ‘Sensation’ peel between 90 and 112 DAFB and continues throughout ‘Guifei’ ripening, these results suggest that the expression of *MiBBXs* may be closely related to anthocyanin synthesis and light exposure (Figure 5a, Supplementary Table S5).

### 3.8. Light-Induced Expression of MiBBXs in ‘Hongmang No. 6’ Peel

**Figure 7:**
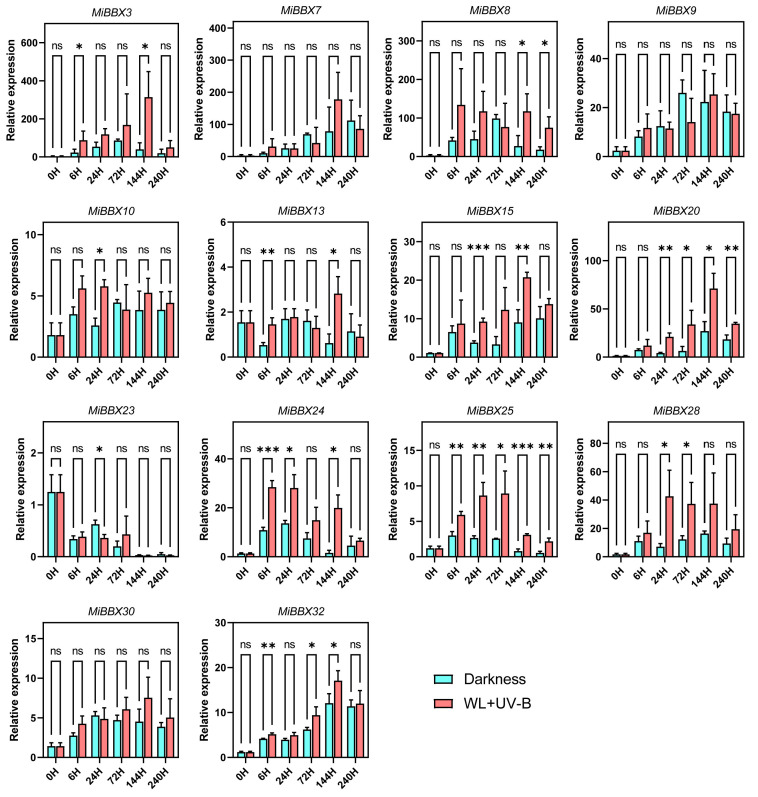
RT-qPCR analysis of *MiBBX* expression in ‘Hongmang No. 6’ at 0, 6, 24, 72, 144, and 240 h after postharvest light treatment. Statistical significance (determined by Student’s *t*-test) is indicated as follows: * *p* < 0.05, ** *p* < 0.01, *** *p* < 0.001, ns *p* > 0.05.

Previous studies have shown that postharvest exposure to white light combined with UV-B radiation promotes anthocyanin biosynthesis in mango peel [47]. RT-qPCR analysis of ‘Hongmang No. 6’ peel samples treated with light at 6, 24, 72, 144, and 240 h post-treatment (HPT) revealed significant induction of multiple *MiBBX* genes, including *MiBBX3*, *MiBBX8*, *MiBBX10*, *MiBBX13*, *MiBBX15*, *MiBBX20*, *MiBBX24*, *MiBBX25*, *MiBBX28*, and *MiBBX32*. For example, *MiBBX20* expression was significantly elevated in treated peel compared to controls at 24, 72, 144, and 240 HPT, while *MiBBX25* was upregulated 1.97-, 3.27-, 3.46-, 3.89-, and 4.02-fold at the respective time points. In contrast, the expressions of *MiBBX7*, *MiBBX9*, *MiBBX23*, and *MiBBX30* were not significantly affected by light treatment (Figure 7).

## 4. Discussion

BBX proteins represent a widely conserved transcription factor family across diverse plant species, playing essential roles in light signaling, development, hormone responses, stress adaptation, and pigment biosynthesis [4,47,50]. Genome-wide studies have identified 32 *BBX* genes in *Arabidopsis* [4], 25 in pear [10], 31 in tomato [51], 59 in soybean [52], 23 in pomegranate [53], 30 in rice [5], and 19 in pineapple [54]. In the present study, we identified 32 BBX genes in mango, a number comparable to that reported in *Arabidopsis* [4]. However, this similarity does not imply conservation, as gene family expansion and diversification are influenced by species-specific evolutionary events.

Gene family expansion in plants typically occurs via whole-genome duplication (WGD), segmental duplication, and tandem duplication events [55]. The mango genome has experienced multiple WGD events, with the most recent estimated at approximately 33 million years ago (MYA) [34], which likely contributed to the expansion and retention of *BBX* genes. Our analysis identified 26 segmentally duplicated *MiBBX* gene pairs, with no evidence of tandem duplication, indicating that segmental duplication has played a predominant role in the expansion of the *BBX* gene family in mango. A similar pattern has been observed in other perennial species such as pineapple [54], pomegranate [53], and *Lagerstroemia indica* [56], suggesting that segmental duplication is a common mechanism driving *BBX* gene expansion in woody plants. Moreover, the Ka/Ks values << 1 for most collinear pairs indicate strong purifying selection [57,58]. This is consistent with the results of most syntenic gene pairs in the mango genome, indicating that MiBBXs tend to become functionally stable after undergoing family expansion events [34].

*BBX* genes often display spatiotemporal and organ-specific expression patterns, reflecting their diverse functions throughout plant development [54]. Our study revealed complex organ-specific expression profiles for *MiBBXs*. Notably, the genes that were highly expressed primarily belonged to subfamilies I, IV, and V, suggesting potential links between evolutionary divergence, expression patterns, and functional specialization [59]. Many *MiBBX* genes showed preferential expression in light-exposed organs such as leaves, bark, flowers, and peel, while the expression was generally lower in roots, seeds, and flesh. These patterns suggest that certain *MiBBX* genes may be responsive to light exposure and potentially associated with light-related developmental or metabolic processes in mango peel, although further functional studies are needed to confirm their roles. Several *MiBBX* genes showed higher expression levels in the peel compared to the flesh of red mango cultivars such as ‘Sensation’ and ‘Guifei’. Additionally, their expression levels were elevated in red-pigmented cultivars compared to yellow or green ones. These trends suggest a possible association between *MiBBX* gene expression and peel pigmentation, potentially through the regulation of anthocyanin biosynthesis, which is known to be strongly influenced by light. Light is a key external signal regulating both *BBX* gene expression and anthocyanin biosynthesis. BBX proteins often act as positive regulators of anthocyanin production either by interacting directly with HY5 or MYB transcription factors or indirectly by modulating *HY5* expression [28,60,61,62,63,64,65]. Several studies have demonstrated the role of BBXs as positive regulators of anthocyanin biosynthesis. For example, in pear, PpBBX16 promotes anthocyanin accumulation under white light; its overexpression in pear calli enhanced red pigmentation under light, whereas virus-induced gene silencing (VIGS) reduced anthocyanin accumulation [28]. Similarly, studies in *Lagerstroemia indica* revealed that most *LiBBX* genes were downregulated in leaves under dark conditions, and their expression levels positively correlated with the anthocyanin and chlorophyll content [56]. Yeast two-hybrid (Y2H) and bimolecular fluorescence complementation (BiFC) assays further demonstrated physical interactions between LiBBX4 and key photomorphogenesis regulators such as LiHY5, LiHYH, and LiCOP [56]. Moreover, VIGS experiments confirmed that LiBBX4 regulates anthocyanin biosynthesis in *Lagerstroemia indica* leaves [56]. Similarly, in poplar, PtrBBX23 directly activates anthocyanin biosynthetic genes and interacts with HY5 [66].

Consistent with these previous findings, we observed that several *MiBBX* genes were more highly expressed in light-exposed peel than in non-exposed flesh in red mango cultivars. Furthermore, our RT-qPCR analysis showed that postharvest light treatment significantly upregulated the expression of 10 *MiBBX* genes (*MiBBX3*, *MiBBX8*, *MiBBX10*, *MiBBX13*, *MiBBX15*, *MiBBX20*, *MiBBX24*, *MiBBX25*, *MiBBX28*, and *MiBBX32*) in the peel of ‘Hongmang No. 6’ at multiple time points. These observations suggest that specific *MiBBX* genes are light-responsive and may play roles in light-induced anthocyanin biosynthesis in mango peel [47]. A promoter analysis further revealed that many of these upregulated *MiBBXs* contained G-box and hormone-responsive *cis*-elements, suggesting their potential involvement in integrating light and hormone signals in the regulation of anthocyanin biosynthesis.

Interestingly, a recent study in mango demonstrated that MiBBX24 (homologous to our MiBBX3) and MiBBX27 (homologous to our MiBBX11) activate *MiMYB* and carotenoid biosynthesis genes (*MiPSY*) under blue light [67], indicating that MiBBXs may regulate not only anthocyanin but also carotenoid biosynthesis pathways. However, whether other *MiBBXs* respond to different light spectra or regulate anthocyanin biosynthesis through distinct mechanisms requires further investigation.

Mechanistically, BBX proteins often function downstream of the key repressor COP1 and upstream of the activator HY5 in the light signaling cascade [22,68,69,70,71], although some *BBXs* are regulated directly by HY5. For example, in *Arabidopsis*, HY5 binds the G-box in the *AtBBX11* promoter, activating its transcription and promoting photomorphogenesis [72], while repressing the negative regulators *AtBBX30* and *AtBBX31* by binding to their promoters [73,74]. The prevalence of G-box elements and hormone-responsive motifs in *MiBBX* promoters supports their potential roles in mediating light and hormonal regulation in mango peel pigmentation.

In summary, our findings suggest that segmental duplication and purifying selection have shaped the evolution of the mango *BBX* gene family, while light-regulated transcriptional control of *MiBBXs* plays a central role in anthocyanin biosynthesis in mango peel. The *MiBBX* genes identified here provide strong candidates for future functional characterization to better understand the regulatory networks controlling fruit pigmentation in mango.

## 5. Conclusions

In this study, we comprehensively identified and characterized 32 *BBX* genes in the mango genome, classifying them into five subfamilies based on conserved domains. Comparative synteny analyses with *Arabidopsis*, citrus, grape, and apple, along with intra-genomic collinearity, revealed that segmental duplication has been the primary driver of *BBX* gene family expansion in mango, with purifying selection contributing to their evolutionary conservation. A promoter analysis demonstrated the widespread presence of light- and hormone-responsive *cis*-elements, suggesting that *MiBBXs* integrate environmental and hormonal signals. Organ-specific expression patterns indicated that many *MiBBX* genes are preferentially expressed in light-exposed organs, particularly the peel, implicating them in light-regulated physiological processes. Notably, fourteen *MiBBX* genes exhibited higher expression in the peel of red cultivars compared to flesh and to non-red cultivars, while ten genes were significantly upregulated in response to postharvest light exposure. These findings suggest that specific *MiBBX* genes participate in light-induced anthocyanin biosynthesis in mango peel. Overall, this work provides valuable insights into the molecular mechanisms underlying light-mediated pigment biosynthesis in mango and establishes a foundation for future functional studies on *MiBBX* genes aimed at improving fruit coloration and quality.

## Data Availability

The data are contained within this manuscript and the Supplementary Materials.

## Supplementary Materials

The following supporting information can be downloaded at https://www.mdpi.com/article/10.3390/biology14080919/s1: Supplementary Figure S1: The phylogenetic tree of the BBX family constructed by the maximum (ML) likelihood method; Supplementary Figure S2: Inter-species collinearity plots; Supplementary Table S1: RT-qPCR primer information; Supplementary Table S2: Physical and chemical characteristics of MiBBX family member proteins; Supplementary Table S3: Ka/Ks ratios and occurrence times of segmentally duplicated *BBX* gene pairs of mango–self, mango–*Arabidopsis*, mango–orange, mango–apple, and mango–grape; Supplementary Table S4: The TPM values of *MiBBXs* in the peel and pulp tissues of ‘Sensation’ at 94, 100, 106, and 112 DAFB; Supplementary Table S5: The TPM values of *MiBBXs* in the rind tissues of ‘Sensation’ (red), ‘Hongyu’ (yellow), and ‘Gure-82’ (green) in the dark-green ripe, moderately ripe, and fully ripe fruit.
