# Supplementary figures and images for: Genome-Wide Identification, Characterization, and Expression Analysis of BBX Genes During Anthocyanin Biosynthesis in Mango (Mangifera indica L.)
Source: Biology (Basel). 2025 Jul 23;14(8):919. doi: 10.3390/biology14080919 (PMC12383879; doi:10.3390/biology14080919)

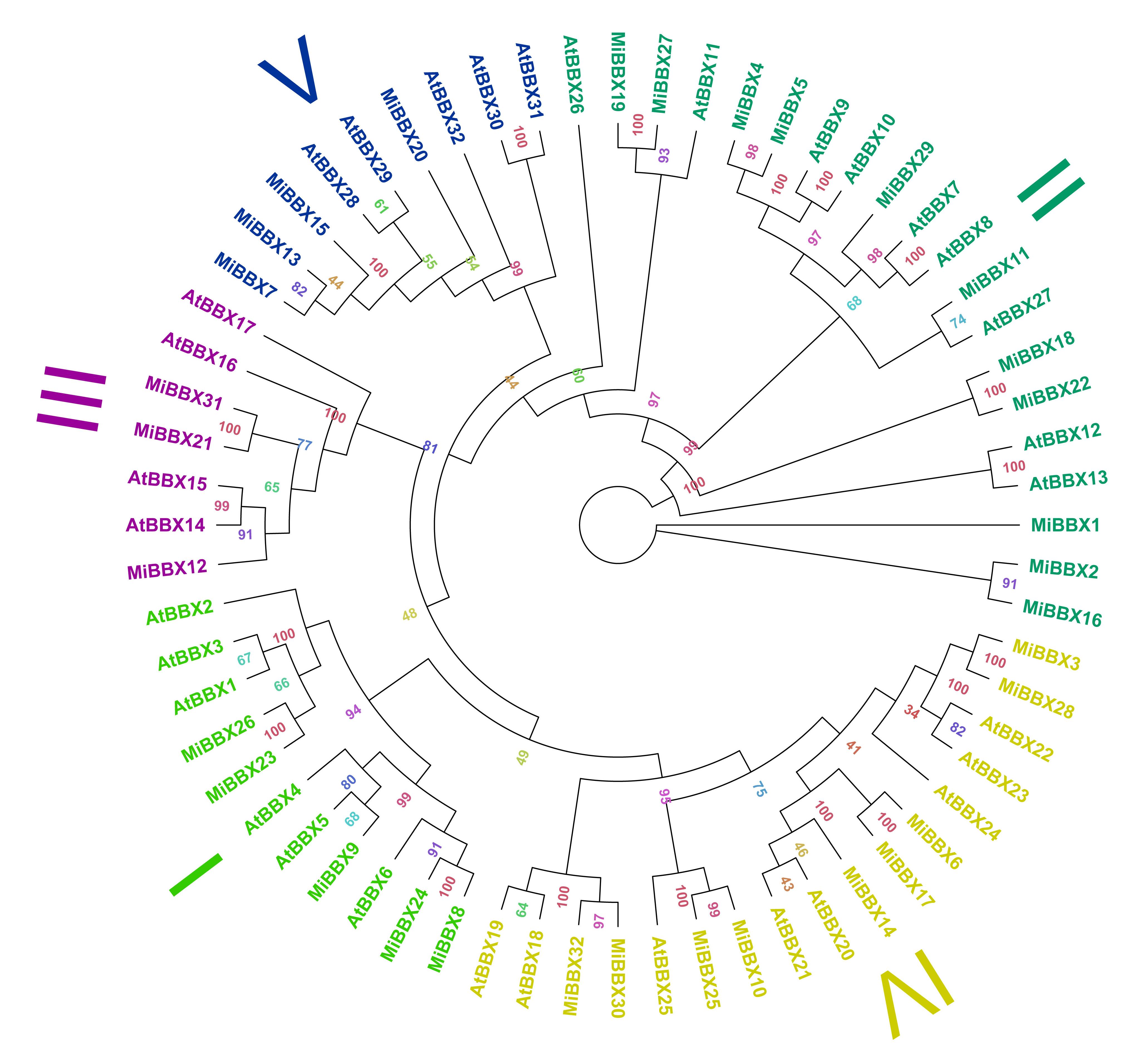

Supplement: Supplementary file 1 [file biology-14-00919-s001.zip › Supplementary Figure S1 The phylogenetic tree of the BBX family constructed by the maximum (ML) likelihood method.jpg]

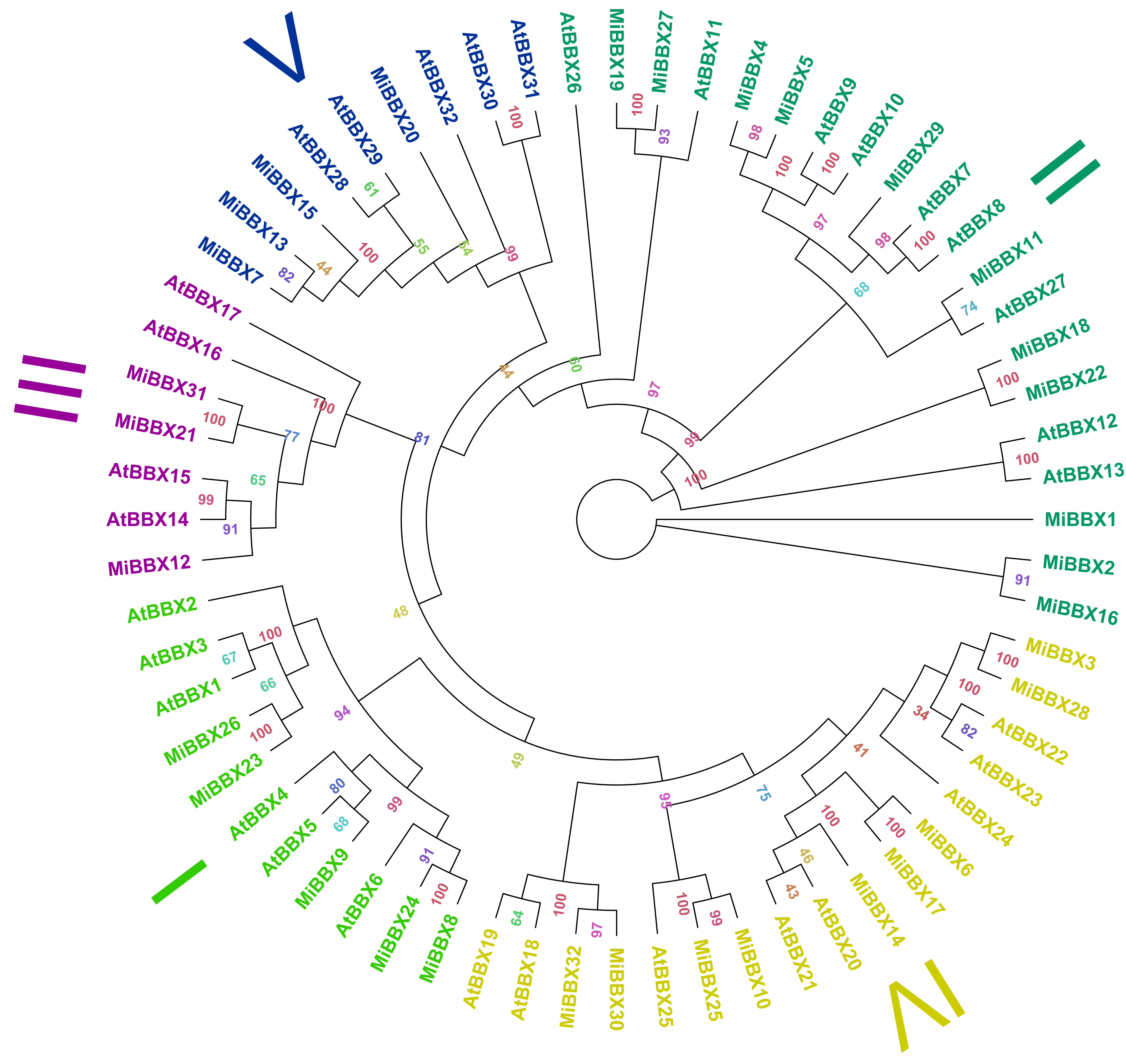

Supplement: Supplementary file 1 [file biology-14-00919-s001.zip › Supplementary Figure S1 The phylogenetic tree of the BBX family constructed by the maximum (ML) likelihood method.tif]

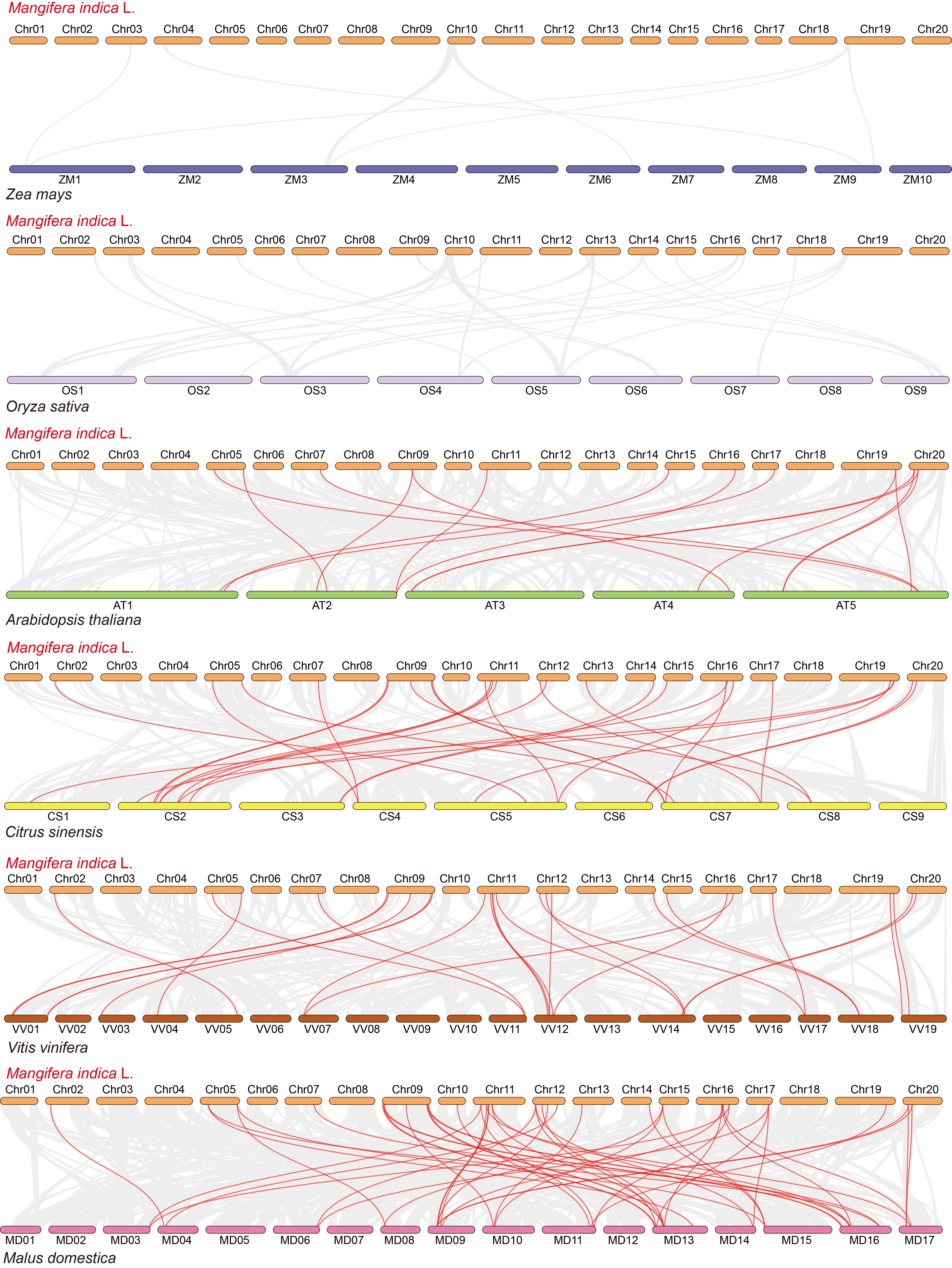

Supplement: Supplementary file 1 [file biology-14-00919-s001.zip › Supplementary Figure S2 Inter-species collinearity plots.jpg]

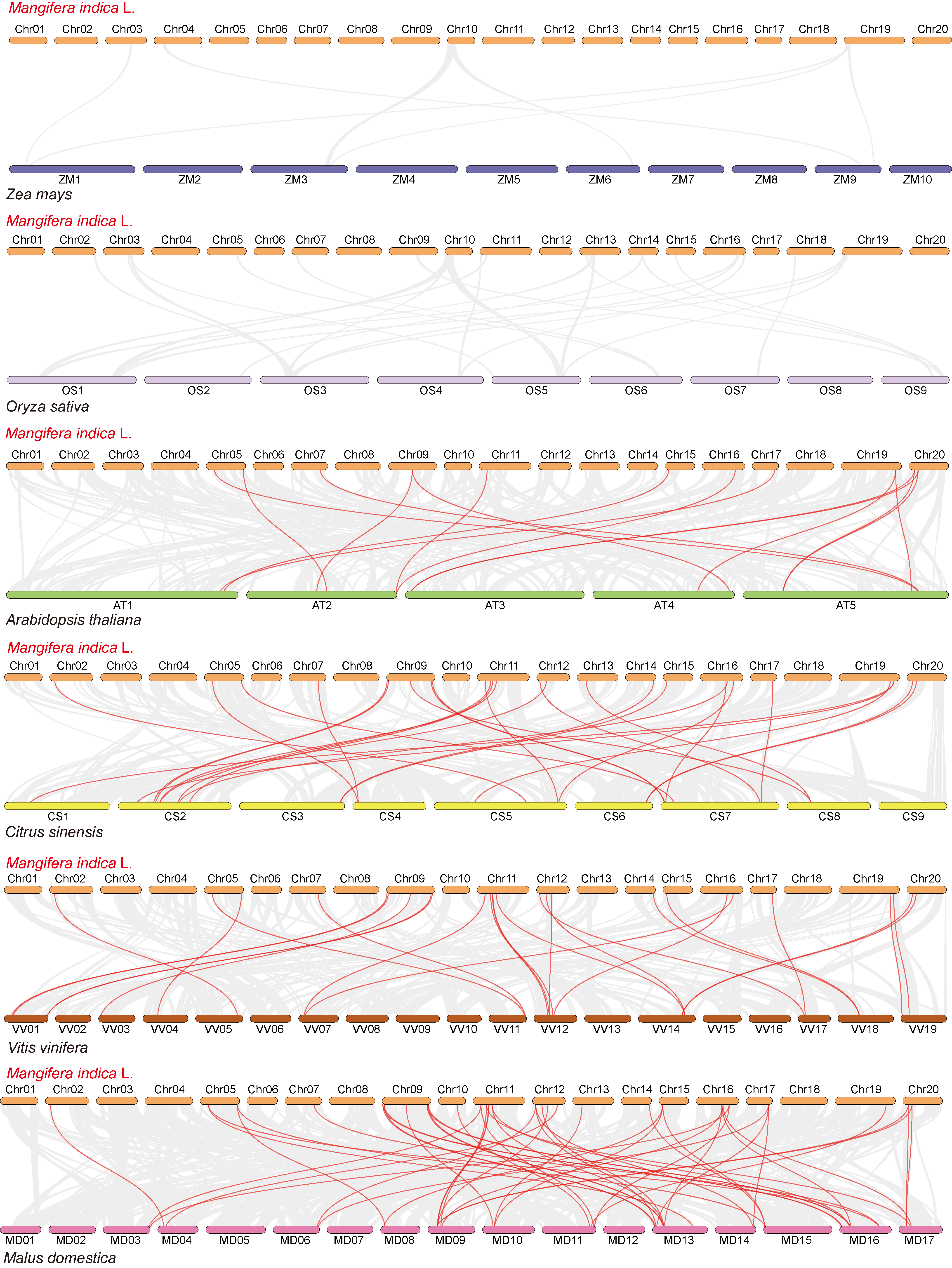

Supplement: Supplementary file 1 [file biology-14-00919-s001.zip › Supplementary Figure S2 Inter-species collinearity plots.tif]
